# High fat diet alters gut microbiota but not spatial working memory in early middle-aged Sprague Dawley rats

**DOI:** 10.1371/journal.pone.0217553

**Published:** 2019-05-29

**Authors:** Nikita Girish Deshpande, Juhi Saxena, Tristan G. Pesaresi, Casey Dylan Carrell, Grayson Breneman Ashby, Min-Ken Liao, Linnea Ruth Freeman

**Affiliations:** 1 Neurosciences, Furman University, Greenville, South Carolina, United States of America; 2 Department of Biology, Furman University, Greenville, South Carolina, United States of America; University of Bonn, GERMANY

## Abstract

As the global population ages, and rates of dementia rise, understanding lifestyle factors that play a role in the development and acceleration of cognitive decline is vital to creating therapies and recommendations to improve quality of later life. Obesity has been shown to increase risk for dementia. However, the specific mechanisms for obesity-induced cognitive decline remain unclear. One potential contributor to diet-induced cognitive changes is neuroinflammation. Furthermore, a source of diet-induced inflammation to potentially increase neuroinflammation is via gut dysbiosis. We hypothesized that a high fat diet would cause gut microbe dysbiosis, and subsequently: neuroinflammation and cognitive decline. Using 7-month old male Sprague Dawley rats, this study examined whether 8 weeks on a high fat diet could impact performance on the water radial arm maze, gut microbe diversity and abundance, and microgliosis. We found that a high fat diet altered gut microbe populations compared to a low fat, control diet. However, we did not observe any significant differences between dietary groups on maze performance (a measure of spatial working memory) or microgliosis. Our data reveal a significant change to the gut microbiome without subsequent effects to neuroinflammation (as measured by microglia characterization and counts in the cortex, hippocampus, and hypothalamus) or cognitive performance under the parameters of our study. However, future studies that explore duration of the diet, composition of the diet, age of animal model, and strain of animal model, must be explored.

## Introduction

The prevalence of obesity among US adults is above 36%, and has been rising significantly since the 1980s [1]. Diet is a significant lifestyle factor in the development of diabetes, hypertension, heart disease, and obesity. The Western Diet is characterized by high saturated fat, cholesterol and simple sugar content [2–4]. Alternatively, diets including fish, n-3 fatty acids, and a high ratio of polyunsaturated fatty acids to saturated fatty acids are associated with decreased risk for developing dementia [5]. Both epidemiological studies in humans and animal studies have begun to elucidate the potential effects of diet on cognition, and the mechanisms through which these effects occur. A comprehensive review of epidemiological studies of fat intake and cognition found support for the hypothesis that diets high in saturated fatty acids and trans-fatty acids are associated with higher risk of cognitive decline and dementia, whereas diets high in polyunsaturated fats or monounsaturated fats are associated with decreased risk for dementia [6].

Obesity at midlife has been correlated with increased risk of development of dementia in humans [7]. Normative aging involves increased risk of cognitive impairment, but consumption of the Western diet may exacerbate this cognitive impairment. Animal models suggest that a high fat diet significantly affects hippocampal-dependent memory and learning, indicating it may be a factor in the risk of dementia development [2, 3, 8, 9]. However, the specific mechanisms through which this influence occurs remain unclear.

Understanding the mechanism by which a high fat diet alters neural environments resulting in cognitive decline is important in establishing targets for potential therapies or treatment. Studies have shown that gut microbiota play a significant role in the development of metabolic syndromes, and gut microbiota are greatly influenced by an obesogenic diet [10]. The colonic microbial community is the most diverse among the gastrointestinal tract, containing almost 400 different species of bacteria [11]. Approximately 90% of these species reside within the Bacteroidetes and Firmicutes phyla with smaller contributions from the Actinobacteria and Proteobacteria phyla, and many of these species are novel and currently unable to be cultured ex vivo [12–14]. The relative abundance and diversity of bacteria within the microbiome has been shown to differ drastically from host to host, depending on host habitat factors like pH, gas availability, and nutrients [11, 15]. Other host attributes like exercise, diet, and health status can also influence the structure of colonic microbial communities. Previous research shows a correlation between obesity and changes in colonic bacterial communities, leading to a dysbiosis of the gut microbiota [14, 16]. This dysbiosis is typically associated with a change in the proportion of the Firmicutes and Bacteroidetes, with the former increasing and the latter decreasing, relative to lean individuals. If persistent, this dysbiosis can ultimately cause systemic low-grade inflammation, which has been shown to play a part in metabolic disorders like type 2 diabetes [17, 18]. Though this change in relative abundances has been associated with an increased likelihood for obesity, this trend can also be reversed through a change in diet [19].

The aims of this study were three-fold: 1) to determine the effects of a high fat diet on spatial working memory as measured by the water radial arm maze in middle-aged Sprague Dawley rats, 2) to evaluate the effects of a high fat diet on the abundance of and diversity of gut bacteria, and 3) to evaluate microgliosis as an indicator of neuroinflammation. We hypothesized that a high fat diet would lead to cognitive decline due to diet-induced gut dysbiosis and subsequent neuroinflammation. Interestingly, we did not find a statistically significant difference in water radial arm maze performance or microgliosis between middle-aged Sprague-Dawley rats fed a low-fat (control) or high-fat diet. However, we did determine significant changes to the gut microbiome. Therefore, these data indicate the possibility of gut dysbiosis without significant cognitive decline or neuroinflammation under the parameters of this study.

## Materials and methods

### Animals and diets

Male Sprague-Dawley rats (Envigo, Prattville, Alabama) were housed in the vivarium one to a cage under a controlled, reverse 12 h light/12 h dark cycle with ad libitum access to food and water. Early middle-aged rats (7 months old; n = 6 per group) were randomly assigned to their dietary group at the start of the study. The rats were fed either a high fat diet (20% protein, 35% carbohydrate, 45% fat by calories; Research Diets #D12451) or control, low fat diet (20% protein, 70% carbohydrate, 10% fat by calories; Research Diets #D12450H) for eight weeks (Research Diets Inc., New Brunswick, NJ). The high fat diet delivers 4.73 kilocalories per gram and the low fat (control diet) which is matched to the high fat diet for sucrose content (17% sucrose) delivers 3.85 kilocalories per gram. The rats underwent cognitive assessment during the last 2 weeks of dietary treatment. Animal protocols were approved by the Furman University Animal Care Committee and carried out according to guidelines from the National Institute of Health. Body weights and food consumption were measured every week throughout the study. Fecal samples were collected at the conclusion of the study using aseptic technique and stored in a -80°C freezer for further analysis.

### Cognitive assessment

An eight-arm water radial arm maze (WRAM) was used to assess working and reference spatial memory in accordance with previously published protocols from similar experiments [8, 20]. Escape platforms were placed in four arms of the maze; the baited arms were randomly assigned for each rat and kept consistent over the 12 days of testing. Spatial cues were positioned around the room, a necessary component to the design of this study. Each of the four areas of the room contained a different cue: A posterboard with black writing on it, a large piece of paper with polka dots, a large piece of paper with diagonal lines, and the experimenter (positioned at the start arm every day and every trial). Other than a sink in the room, there were no other cues and white walls behind the cues described above. Every day, four trials (maximum of three minutes each) were administered for each rat. After each trial, the successfully-located platform (or, in the event of a failure, the nearest platform at the end of the three minutes) was removed, thus establishing a win-shift paradigm for the task. Three types of errors were quantified: Working Memory Correct (WMC), Reference Memory (RM), and Working Memory Incorrect (WMI). WMC errors were first and repeat entries into an arm that previously contained a platform. RM errors were first entries into any arm that never contained a platform. WMI errors were repeat entries into an arm that never contained a platform (i.e. repeat entries into a RM arm). Prior to testing, all rats were evaluated in the maze for swim speed and vision by utilizing a flagged platform. All animals met the criteria for swimming and vision abilities. Trial 1 WMC errors were not included in the analysis because it is not possible to make a WMC error on the first trial of each day.

### Fecal sample analysis

Fecal samples were sent for next generation sequencing and genus analysis (MR DNA Labs, Shallowater, TX). For this analysis, DNA from approximately 200 mg of each fecal sample was extracted using the PowerSoil DNA Isolation Kit (Qiagen, Redwood City, CA), and the extracted DNA was amplified via endpoint PCR using the 16S universal Eubacterial primers 515F GTGCCAGCMGCCGCGGTAA and 806R GGACTACHVGGGTWTCTAAT. The amplified DNA was sequenced using bacterial tag-encoded FLX amplicon pyrosequencing as described by Dowd et al. 2008 [21]. Genera abundances were reported as percentages of total bacteria, and diversity analysis was completed using Qiime (qiime.org).

### Immunohistological evaluation

After rats were anesthetized deeply with isoflurane gas, brains were rapidly removed. The right hemisphere was drop-fixed in 4% paraformaldehyde for 48hr, and then stored in 30% sucrose in phosphate buffered saline at 4°C for later evaluation of microglia. Coronal sections (40 um) were processed using standard immunohistochemistry procedures with Iba-1 (FUJIFILM Wako Pure Chemicals, Richmond, VA, 1:1,000). Free-floating sections were incubated overnight at 4°C with primary antibody, and then incubated with secondary antibody for 2hr at room temperature. Sections were washed and placed in Elite ABC reagent (Vector, Burlingame, CA), and then washed and incubated with a Vector VIP Kit (Vector, Burlingame, CA). Finally, sections were washed, mounted, and coverslipped with Vectamount. To avoid group inter-variability in staining, sections from both dietary groups were incubated in the same bath. Hippocampal, hypothalamic, and cortical images were captured using an EVOS fi-AMG light microscope. Researchers blind to the groups characterized and counted microglia in at least three sections per animal. Microglia were characterized as ramified (resting) if they exhibited a small cell body and long, thin processes. Microglia were characterized as activated if they exhibited a large cell body, shorter processes, and greater immunoreactivity compared to resting microglia.

### Statistical analysis

Results are expressed as mean ± SEM. All statistical analyses were performed using GraphPad Prism 8.0 software (GraphPad Software, La Jolla, CA, USA). Water radial arm maze performance was analyzed using a two-way ANOVA in order to evaluate diet x days main effects and interaction effects. Body weights and food consumption were also analyzed using a two-way ANOVA in order to evaluate diet x week main effects and interaction effects. Microglial cell counts were analyzed using a Student’s t-test. Statistical analysis for diversity in gut bacteria is described in more detail in the results section.

## Results

### Food consumption and change in body weight

**Fig 1:**
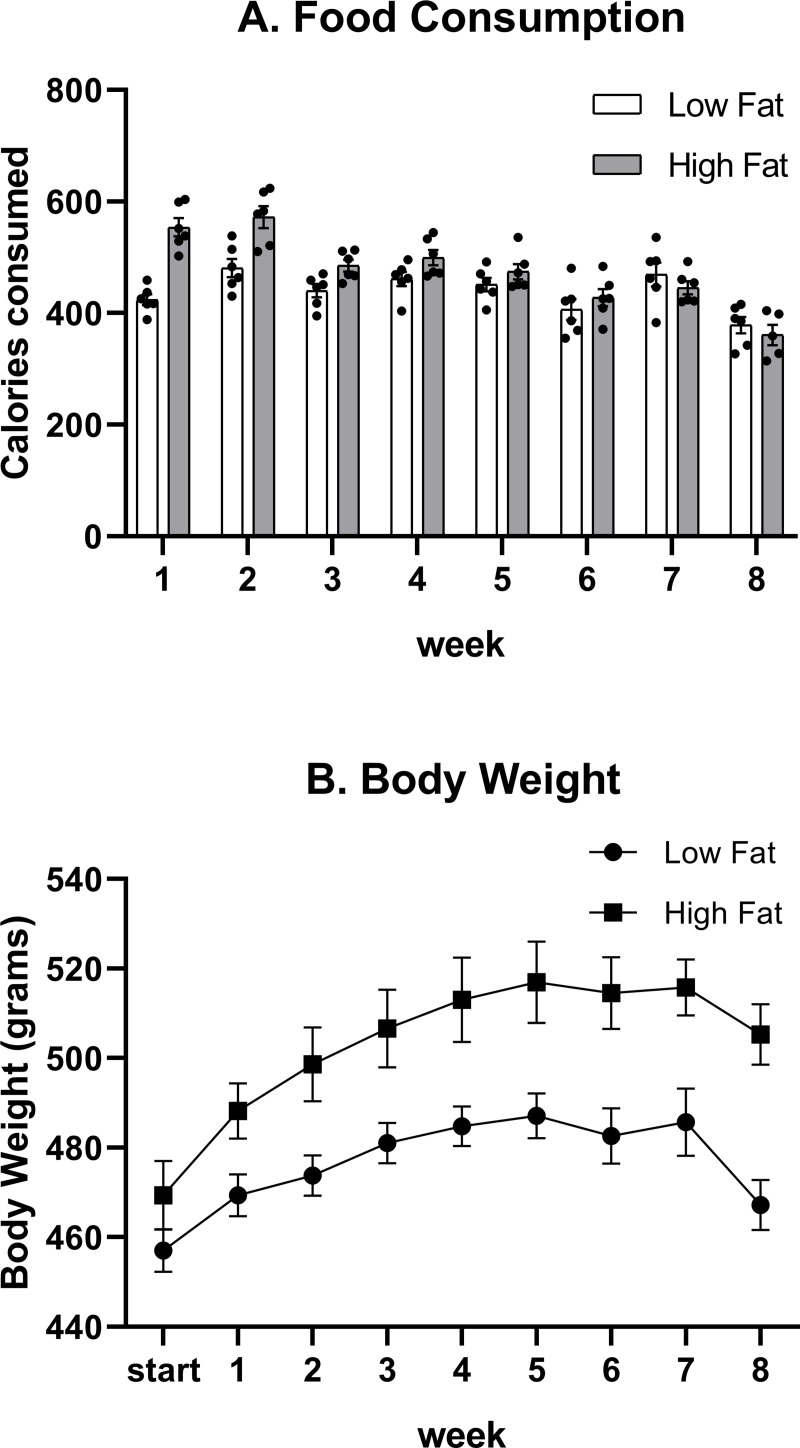
Food consumption and body weight. Food consumption was significantly different between low fat and high fat-fed rats over the 8 weeks. Body weights at the start of the study were not significantly different. Once dietary treatment began, body weights were significantly different between groups throughout the study.

Food consumption was evaluated weekly and then normalized for calories consumed by dividing grams consumed per week by kilocalories per gram specific to the diet. The high fat diet provides 4.73 kilocalories per gram while the control, low fat diet provides 3.85 kilocalories per gram. A main effect of diet (F_(1,10)_ = 5.820, p = 0.0365), a main effect of weeks (F_(7,69)_ = 38.76, p <0.0001) and an interaction effect of diet x week (F_(7,69)_ = 12.04, p<0.0001) were determined for food consumption (Fig 1A). At the start of the study, there was no significant difference in body weights between groups (Low Fat: 457.03 ± 11.67 grams, High Fat: 469.4 ± 18.9 grams, p = 0.202). A main effect of diet (F_(1,10)_ = 9.016, p = 0.0133), a main effect of week (F_(8,80)_ = 45.91, p<0.0001), and an interaction effect of diet x week (F_(8,80)_ = 3.980, p = 0.0005) were determined for body weight (Fig 1B).

### Diversity in gut bacteria

After stringent quality sequence curation, a total of 1,318,811 sequences were parsed and 1,122,322 were then clustered. 1,122,297 sequences identified within the Bacteria and Archaea domains were utilized for final microbiota analyses. The average reads per sample was 93,524. For alpha and beta diversity analysis, samples were rarefied to 30,000 sequences and bootstrapped at 25,000 sequences. Weighted UniFrac distance matrices were used to compare the beta diversity of the high fat and control diet-fed gut microbial communities and showed significantly (p = 0.005) different phylogenetic assemblages between the two groups after the diet (Table 1). Statistical comparisons were conducted using ANOVA, and post-hoc pairwise comparisons were made using Tukey’s test. We evaluated whether any specific genera were significantly different between treatment groups. There were a wide range of genera found to be significantly different between groups. Table 1 displays the genera that had significantly different relative abundances based on dietary group.

**Table 1:** Diversity in gut microbes. Mean relative abundances for gut genera and p-value for differences between high-fat and control animals are listed. Class and phylum are indicated as well. This table reveals genera that were determined to be significantly different between dietary groups. Genera that are not significantly different are not listed due to the large number of microbes evaluated.

*Genus*	Class	Phylum	Mean Relative Abundance: Low Fat	Mean Relative Abundance: High Fat	Effect of high fat diet	p-value
*Granulicatella*	Bacilli	Firmicutes	0.001	0.004	Increase	0.012
*Lactococcus*	Bacilli	Firmicutes	0.549	1.252	Increase	0.013
*Anaerobacterium*	Clostridia	Firmicutes	0.001	0.005	Increase	0.003
*Anaerovorax*	Clostridia	Firmicutes	0.010	0.025	Increase	0.028
*Clostridium*	Clostridia	Firmicutes	12.598	22.439	Increase	0.003
*Coprococcus*	Clostridia	Firmicutes	0.105	0.330	Increase	0.042
*Eubacterium*	Clostridia	Firmicutes	3.719	5.351	Increase	0.017
*Faecalibacterium*	Clostridia	Firmicutes	0.107	0.386	Increase	0.033
*Flavonifractor*	Clostridia	Firmicutes	0.009	0.014	Increase	0.031
*Oscillospira*	Clostridia	Firmicutes	0.966	1.704	Increase	0.013
*Papillibacter*	Clostridia	Firmicutes	0.071	0.120	Increase	0.035
*Pseudoflavonifractor*	Clostridia	Firmicutes	0.447	1.314	Increase	0.003
*Robinsoniella*	Clostridia	Firmicutes	2.688	0.109	Decrease	0.002
*Sporobacter*	Clostridia	Firmicutes	0.026	0.045	Increase	0.016
*Tyzzerella*	Clostridia	Firmicutes	0.078	0.160	Increase	0.037
*Erysipelatoclostridium*	Erysipelotrichia	Firmicutes	0.036	0.096	Increase	0.035
*Holdemania*	Erysipelotrichia	Firmicutes	0.004	0.008	Increase	0.035
*Bacteroides*	Bacteroidia	Bacteroidetes	9.973	5.937	Decrease	0.029
*Barnesiella*	Bacteroidia	Bacteroidetes	4.572	2.676	Decrease	0.021
*Rothia*	Actinobacteria	Actinobacteria	0.044	0.078	Increase	0.013
*Adlercreutzia*	Coriobacteriia	Actinobacteria	0.011	0.060	Increase	0.015
*Collinsella*	Coriobacteriia	Actinobacteria	0.001	0.004	Increase	0.008
*Citrobacter*	Gammaproteobacteria	Proteobacteria	0.036	0.003	Decrease	0.017

While there was a significant difference found in the number of observed species between treatment groups (alpha diversity; p = 0.016), once both richness and evenness were accounted for using the Shannon diversity metric, no significant difference was found (p = 0.109). Statistical comparisons were conducted using Kruskal-Wallis test, and Steel-Dwass post-hoc for multiple pairwise comparisons. The observed taxonomic unit counts were evaluated for each of the groups along with the Shannon index.

### WRAM behavior

**Fig 2:**
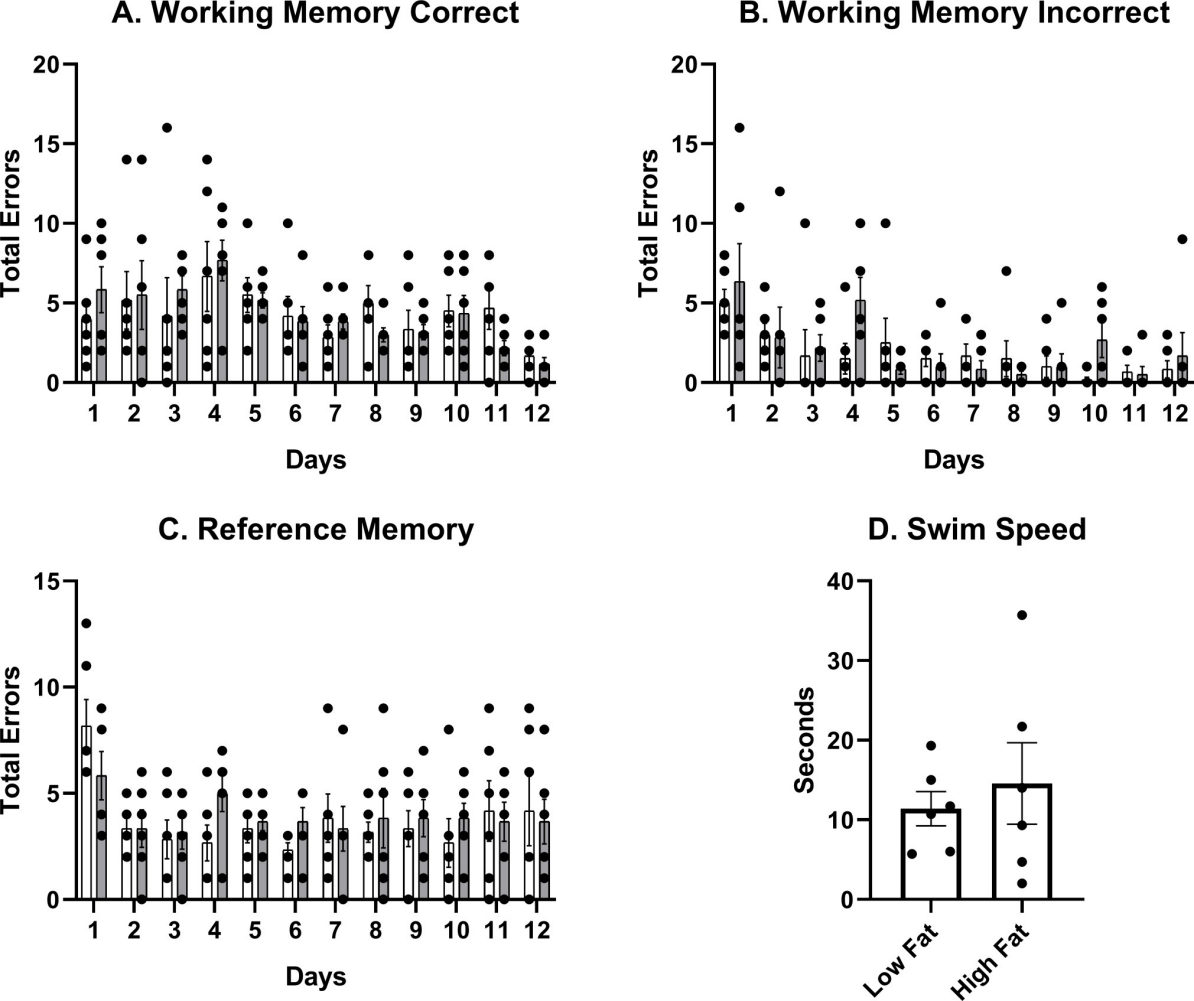
Water radial arm maze performance. Working memory correct (A), working memory incorrect (B) and reference memory (C) errors over the 12 days of testing are shown between low fat and high fat-fed rats. There were no significant differences between dietary group for any of the errors. There were no significant differences in swim speed, as measured by a pre-test (D).

Low fat and high fat diet-fed rats were compared across the 12 days of testing for three different types of errors: working memory correct (WMC), working memory incorrect (WMI), and reference memory (RM). A significant decrease in errors across days was observed for all three errors demonstrating learning during the task, regardless of dietary treatment (WMC: F_(11,110)_ = 2.653, p = 0.0047, Fig 2A; WMI: F_(11,110)_ = 3.387, p = 0.0005, Fig 2B; RM: F_(11,110)_ = 2.722, p = 0.0038, Fig 2C). Furthermore, there were no significant differences in swim speed and vision as determined by a pre-test (p = 0.579). During the pre-test, rats were timed for their swim speed to reach a flagged platform in the maze on the day before testing began. Three trials were conducted; the average of the three trials for each rat (n = 6) are shown in Fig 2D. No significant differences between groups were determined for any of the types of errors (WMC: F_(1,10)_ = 0.0008, p = 0.9780, Fig 2A; WMI: F_(1,10)_ = 0.9838, p = 0.3447, Fig 2B; RM: F_(1,10)_ = 0.1529, p = 0.7040, Fig 2C). The first and fourth trial on each of the 12 days of testing was also evaluated. The first trial was evaluated for each day in order to determine rats’ spatial memory between days and to measure improvements made when working memory load is low. The fourth trial has the highest working memory load, therefore, we determined whether improvements were made between days at the highest level of difficulty as well. No significant effect of diet (WMI: F_(1,10)_ = 0.06421, p = 0.8051; RM: F_(1,10)_ = 0.06453, p = 0.8046) was determined on trial 1 for WMI and RM errors. However, a significant effect of days reveals improvement on trial 1 over time (WMI: F_(11,110)_ = 4.205, p<0.0001; RM: F_(11,110)_ = 4.075, p<0.0001). By definition, it is not possible for a rat to make a WMC error on the first trial, each day. We compared WMC trial 2 between diets, across days and found no significant effect of diet (F_(1,10)_ = 0.000, p>0.999), but a significant effect of days (F_(11,110)_ = 2.148, p = 0.0225). No significant effect of diet (WMC: F_(1,10)_ = 0.6749, p = 0.4305; WMI: F_(1,10)_ = 0.5263, p = 0.4848; RM: F_(1,10)_ = 0.9791, p = 0.3458) or days (WMC: F_(11,110)_ = 1.491, p = 0.1448, WMI: F_(11,110)_ = 0.9742, p = 0.4741; RM: F_(11,110)_ = 0.7289, p = 0.7088) was determined for each of the types of errors on trial 4. While rats learned during this task, as shown by a significant decrease in total errors for each error type and the first trial errors for each error type, they did not significantly decrease errors during trial 4, and this was independent of dietary treatment.

### Microglia

**Fig 3:**
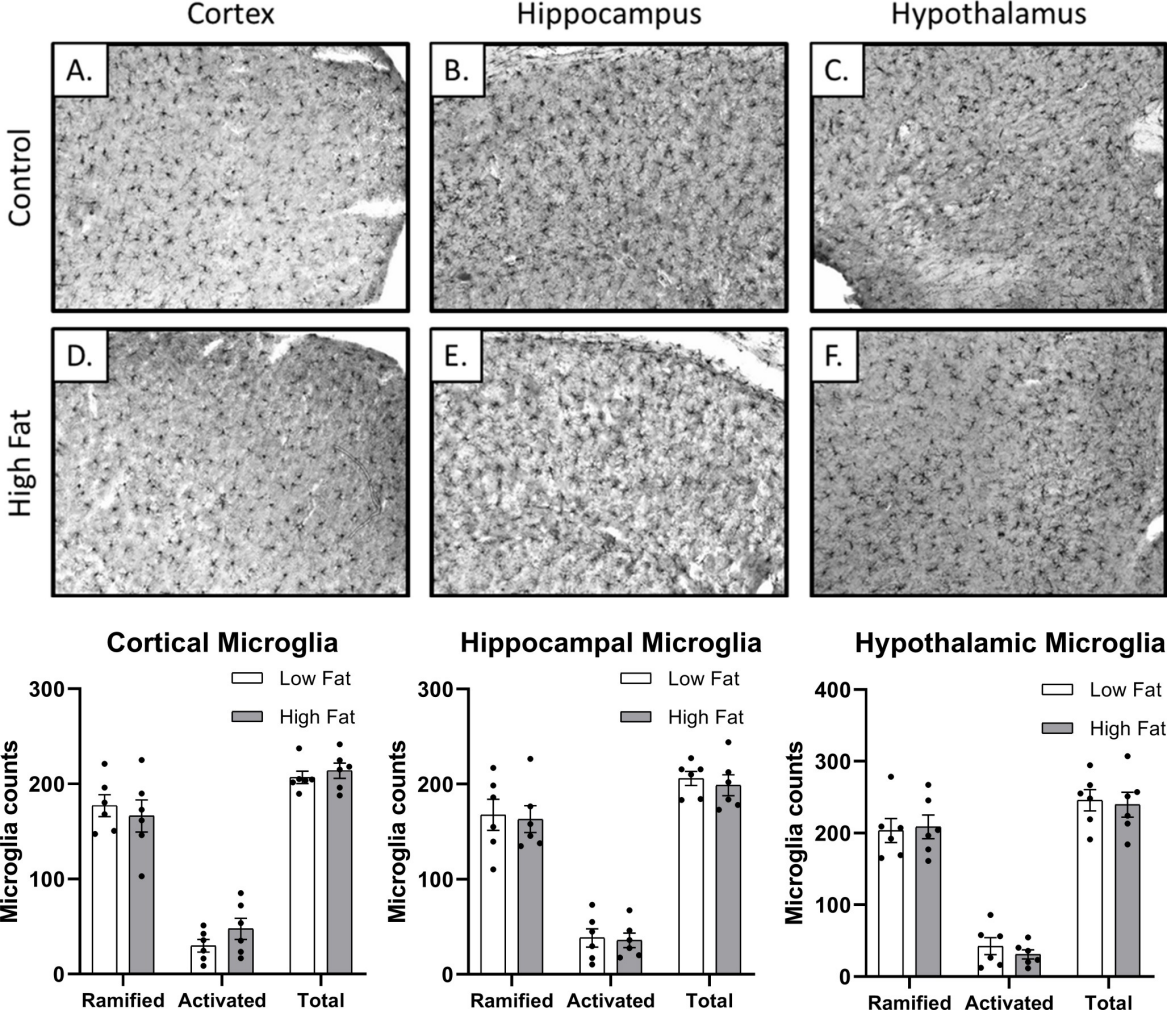
Microglial counts in cortex, hippocampus, and hypothalamus. Fig A-C reveals representative images for low fat-fed (control) rats. Fig 3D–3F reveals representative images for high fat-fed rats. Activated microglia and ramified (resting) microglia were characterized and counted by a researcher blind to the groups. No significant differences in activated, ramified, or total microglia were found between the two dietary groups.

No statistically significant differences (p>0.05) in the number of ramified (resting) or activated microglia were observed in any of the brain regions evaluated between control and high-fat fed rats (Fig 3). The neocortex and dorsal hippocampus were evaluated due to their role in learning and spatial memory. The hypothalamus was evaluated due to its role in feeding, metabolism, and homeostasis.

## Discussion

The current study measured the change in gut microbes following consumption of a high-fat or control diet, performance on the water radial arm maze, and Iba-1 immunohistochemistry in three relevant brain regions. Contrary to our hypothesis, we observed changes to gut microbe populations without subsequent effects to neuroinflammation or working memory behavior.

Previous studies have also shown relationships between high-fat diet, obesity, and dysbiosis in the relative abundances of Firmicutes and Bacteroidetes [12, 14, 16]. Additionally, previous studies have shown changes to cognition following consumption of a high fat diet [8, 9, 20, 22, 23].

More studies linking high fat diet-induced gut dysbiosis and its role in cognition are beginning to emerge. Our study demonstrates diet-induced gut dysbiosis without working memory deficits under the parameters of this study. It is important to highlight that the current study utilized 7-month old male Sprague Dawley rats fed the high-fat or control (low-fat, matched sucrose content) diet for 8 weeks. In a previous study from our laboratory, we fed 16-month old Fisher 344 rats a high saturated fat (10% hydrogenated coconut oil) or control (12% soybean oil) diet for 8 weeks. Using this diet, no significant differences in body weight or food consumption were observed during the 8-week dietary treatment. However, rats fed the saturated fat diet made significantly more WMC and WMI errors during the asymptotic phase (days 7–12) of the water radial arm maze. Saturated fat-treated rats also exhibited greater microglial activation in the hippocampus compared to controls [20]. In another previous study from our laboratory, we fed young (4-month old) and aged (14-month old) Fischer 344 rats a high fat or chow diet for 6 months. Both young and aged rats exhibited worse performance on the 12-day water radial arm maze following consumption of the high fat diet as well as increased microglial activation in the hippocampus for aged rats [8]. Unfortunately, we did not evaluate gut microbe dysbiosis during either of these previous studies. The design of the current study included an early middle-aged rat fed a high fat diet or low fat diet for 8 weeks; the dietary treatment duration was the same as our 2008 study given the previously-observed significant effects on the WRAM. However, we were interested in utilizing a better-designed diet as diet formulations have improved over the last decade. Therefore, we employed the diet-induced obesity (DIO) series from Research Diets, including an appropriate control diet matched for sucrose content, but lower in fat in order to test effects of fat on our experimental outcomes. Furthermore, we utilized the outbred strain, Sprague-Dawley rat, versus the inbred strain, Fisher 344 rat. Sprague-Dawley rats are a multipurpose model, and have been used in other DIO studies, therefore supporting our choice in using this strain. Lastly, we evaluated fecal microbe populations for every rat, providing novelty to this study in that diet-induced effects on gut, brain, and behavior were evaluated for each rat. However, we were surprised that the diet did not alter WRAM performance or microglial activation as we found in our previous studies. A lack of effect could be due to rat strain, diet composition, age of rat, or a combination of these factors. Future studies will be necessary to determine which factor(s) influenced our current lack of results for WRAM and microglial activation.

Although we did not observe differences in WRAM performance, we were intrigued to find changes to the gut microbiome without changes to behavior. In 2015, Magnusson et al. found significant alterations to the gut microbiome following consumption of a high fat diet or a high sucrose diet compared to a chow diet after just two weeks. Two-month old C57BL/6 mice were fed their diets for a total of 6 weeks and behavioral testing was performed throughout the study. Mice fed the high sucrose diet exhibited impairments in the probe trial on the first day of Morris Water Maze testing, indicating some changes to cognitive flexibility and memory. However, there was no significant effect of diet on any of the other behavioral tests performed in this study, including: step-down latency and open field exploration, novel object recognition, and the other trials for the Morris Water Maze [24]. Therefore, we are providing additional support for changes to the gut microbiome without changes to spatial working memory. However, our study only focused on high fat versus low fat and Magnusson’s study evaluated high sucrose versus low sucrose and high fat versus chow.

In an elegant study by Bruce-Keller et al., 3-month old male C57BL/6 mice received donor microbiota via oral gavage from mice either fed a high fat diet or low fat diet for 10 weeks. The high fat diet utilized in the study by Bruce-Keller et al. was Research Diets D12492 which delivers 60% kilocalories from fat versus D12491 utilized in our study which delivers 45% kilocalories from fat. Furthermore, in the study by Bruce-Keller et al., a regular chow diet (Purina Lab Diet 5001) was administered to controls. Mice that received the high fat-derived microbiota were impaired on a number of cognitive tests, in the absence of obesity and in the absence of high fat diet consumption. Specifically, mice that received the high fat-derived microbiota revealed increased anxiety and increased stereotypic behavior on the open field test, as well as impaired contextual fear conditioning. Previous studies have shown an important relationship between gut dysbiosis and mood [25–27]. It is possible that gut dysbiosis has a greater influence on systems related to anxiety, fear, and depression, compared to memory, especially spatial memory. Furthermore, data from this study indicated diet-induced alterations to the Firmicute phyla to have the greatest effects on the recipient mice. In particular, *Bilophila sp*. was hypothesized to be an important contributor to endotoxin release following high fat diet consumption. We have also observed diet-induced alterations to the Firmicute phyla in our study, as well as the Bacteroidetes phyla (see Table 1). However, we did not observe diet-induced changes to *Bilophila sp*., specifically (Low Fat relative abundance of *Bilophila sp*.: 0.041 ± 0.031; High Fat relative abundance of *Bilophila sp*.: 0.058 ± 0.028, p = 0.331). Not only were behavioral changes observed in the study by Bruce-Keller et al., the researchers also determined systemic and brain inflammation following high fat-derived microbiota administration, including increased expression of Iba-1, TLR2, and TLR4 in the medial prefrontal cortex [28]. We hypothesized that inflammation would play a role in diet-induced cognitive changes in our experiment. However, under the parameters of our study, we did not observe diet-induced changes to microglial activation. It is possible that our lack of observed changes in microglial activation is due to stress from the WRAM; both groups underwent the stressful 12 days of swimming and memory task which could increase microglial activation independent of dietary treatment. However, in comparison to our previous studies [8, 20] where microglial activation was observed, WRAM testing did not seem to affect this outcome. Instead, the lack of effect seems to be due to the diets administered. Furthermore, it is possible that our lack of inflammatory response, lack of behavior changes, and lack of change to *Bilophila sp*. abundance compared to the study by Bruce-Keller et al. are due to differences in diets utilized.

Previous research from our laboratory and others has shown increased microglial activation in the brain following consumption of a high fat diet [20, 29–32]. Neuroinflammation is a possible mechanism for cognitive decline, linking gut dysbiosis and cognitive impairments following consumption of a high fat diet [2]. We hypothesize that altered bacterial and archaeal populations thriving on a high fat, high sugar diet could lead to increased endotoxin production, subsequent increased circulating inflammatory factors, and subsequent microglial activation. More studies will be necessary to investigate this mechanism. Work from Bruce-Keller et al., Marques et al., and Beilharz et al. have provided a strong foundation. Marques et al. have demonstrated increased expression of inflammatory markers in the cortex as well as decreased BDNF levels in brain and plasma in Wistar rats fed a high fat diet for 17 weeks [33]. As stated in a correspondence to Biological Psychiatry, Marques et al. hypothesizes an initial change in gut microbiota with a subsequent inflammatory response from the gut to the brain [34]. A number of studies by Beilharz et al. have explored the relationship between macronutrients, gut dysbiosis, neuroinflammation, and cognition [35–39]. In a short-term study where rats were exposed to a chow diet, chow + 10% liquid sucrose, or cafeteria diet + 10% liquid sucrose for two weeks, a significant diet-dependent difference in neuroinflammatory markers was determined. Specifically, rats receiving the 10% sucrose solution revealed significantly increased expression of inflammatory genes in both the hippocampus and white adipose tissue (WAT), including interleukin-1 beta (IL-1β) and tumor necrosis factor alpha (TNFα). The observed increase in hippocampal inflammatory markers correlated with blood glucose levels. This effect was only found in rats fed the 10% sucrose solution alone; rats fed the cafeteria diet with 10% sucrose did not reveal significantly elevated inflammatory markers in the hippocampus or WAT. It is important to note that rats that only received 10% sucrose consumed significantly more liquid sucrose compared to animals fed the high fat, high sugar cafeteria diet. Neither treatment led to increased inflammation in the perirhinal cortex or hypothalamus. However, both the cafeteria + sucrose diet and chow + sucrose diet led to hippocampal-dependent memory deficits after just one week on the diet [36]. In a separate study, specific macronutrients were evaluated; a diet high in saturated fat and a diet high in sugar for two weeks resulted in impaired hippocampal-dependent place recognition memory compared to chow-fed and PUFA-fed rats. In this study, no significant differences in mRNA expression of hippocampal or hypothalamic inflammatory markers were determined. However, the diets caused significant changes to the microbiome [39]. In a recent study, Beilharz et al. fed rats the cafeteria diet or control diet (chow) for 25 days and evaluated effects of varying doses of a probiotic, VSL#3. The rats fed the cafeteria diet revealed impaired performance on a hippocampal-dependent task, increased inhibitor of nuclear factor kappa-B kinase subunit beta (IKBKB) and glial fibrillary acidic protein (GFAP) gene expression in the hippocampus, and increased monocyte chemoattractant protein-1 (MCP-1) in the perirhinal cortex compared to control-fed rats. No effect of the diet on anxiety, as measured by elevated plus maze, was determined [35]. Taken together, a series of experiments from Beilharz et al. have determined diet-dependent changes to inflammatory gene expression in the hippocampus, with the greatest effects from a high sucrose diet, not the cafeteria diet. In our current experiment, both groups received the same amount of sucrose which could account for our lack of observed differences in microglial activation, as a measure of neuroinflammation. In all of the studies by Beilharz et al, the high sucrose diet and cafeteria diet resulted in impaired hippocampal-dependent memory, regardless of changes to inflammatory gene expression, and regardless of differences in energy consumption or weight gain. We did not determine differences in our hippocampal-dependent task. It is possible that without comparison to a chow-fed control, since our control diet was matched for sucrose, diet-dependent reduced memory performance could not be determined.

In a recent study by Cope et al., 8-week old male mice were fed a high fat diet (Research Diets #12451), a high sucrose diet, or standard chow. In order to better understand the role of high fat diet-induced microglial activation on cognitive decline, the researchers elegantly utilized three different techniques to block microglial activation: minocycline administration, annexin-V injections, and mice with a partial knockdown of Cx3cr1 (chemokine fractalkine receptor). Both the high fat and high sucrose diets impaired performance on the hippocampal-dependent object location task and increased microglial activation. Interestingly, all three manipulations to microglia resulted in improved performance on the object location task despite being fed a high fat or high sucrose diet. Furthermore, it was hypothesized that synapse loss due to increased activity by microglia contributed to obesity-induced cognitive impairment [31]. This study provides further support for the idea that neuroinflammation, and specifically microglial activation, can influence cognition. In comparison to our study, 8-week old mice were utilized in the study by Cope et al. versus 7-month old Sprague Dawley rats utilized in our study. While we utilized the same high fat diet, Cope et al. utilized the Purina Lab Diet 5001 chow for comparison as well as a group receiving chow and 34% sucrose water.

In conclusion, we determined altered gut microbe profiles following consumption of a high fat diet compared to rats fed a low fat diet (matched for sucrose). We did not observe altered performance on the water radial arm maze or increased microglial activation. However, we continue to hypothesize a role for gut microbe dysbiosis in neuroinflammation and subsequent cognitive decline. Further studies will be necessary to determine whether inflammation and cognitive decline follow gut microbe dysbiosis. Based on recent findings in mice, we continue to hypothesize this order of events.
